# The human olfactory amygdala: Anatomical connections between the olfactory bulb and amygdala subregions

**DOI:** 10.1162/imag_a_00571

**Published:** 2025-05-09

**Authors:** Qiaohan Yang, Guangyu Zhou, Andrew Sheriff, Vivek Sagar, Shiloh Echevarria-Cooper, Gregory Lane, Thorsten Kahnt, Christina Zelano

**Affiliations:** Department of Neurology, Feinberg School of Medicine, Northwestern University, Chicago, IL, United States; National Institute on Drug Abuse Intramural Research Program, Baltimore, MD, United States

**Keywords:** amygdala, olfaction, diffusion-weighted MRI

## Abstract

The human olfactory bulb is thought to send direct monosynaptic projections to the amygdala, though anatomical evidence for this is scant. Here, we applied a specialized diffusion-weighted magnetic resonance imaging protocol optimized for olfactory brain areas to systematically quantify connections between the olfactory bulb and the amygdala in 25 healthy human participants. We found that the olfactory bulb exhibits a higher density of streamline connections to the medial nucleus, the anterior cortical nucleus, the central nucleus, and the periamygdala complex compared to the basomedial nucleus, the basolateral nucleus, the lateral nucleus, and the posterior cortical nucleus. We used k-means clustering algorithms to confirm these results by performing a data-driven grouping of amygdala subregions into those that connect to the olfactory bulb and those that do not. We further found that olfactory amygdala subregions and non-olfactory amygdala subregions exhibit different structural connectivity patterns with the rest of the brain. Our findings provide confirmatory evidence that a set of amygdala subnuclei—medial nucleus, anterior cortical nucleus, central nucleus, and periamygdala complex—communicate with the olfactory bulb and contribute to primary olfactory processing in humans.

## 1 Introduction

Sensory information from all modalities reaches the amygdala, typically after thalamic and primary sensory processing. By contrast, the olfactory system is directly connected to the amygdala, including monosynaptic projections from olfactory bulb (OB) to multiple amygdala subregions. These subregions can be considered part of primary olfactory cortex, together with the anterior olfactory nucleus, olfactory tubercle, and piriform cortex, all of which receive monosynaptic projections from the OB in parallel (Carmichael et al., 1994;Lane et al., 2020). Projections from the olfactory bulb to the amygdala are poorly characterized in humans, but have been well laid out in rodents. Rodent amygdala subregions receiving direct input from the OB include the anterior and posterior cortical nuclei (ACo and PCo), medial nucleus (MeA), nucleus of the lateral olfactory tract, bed nucleus of the accessory olfactory tract, and anterior amygdaloid area (Allison, 1953;Gutiérrez-Castellanos et al., 2010;Kang et al., 2009;Pro-Sistiaga et al., 2007;Santiago & Shammah-Lagnado, 2004). In non-human primates, direct OB projections have been described in the anterior cortical amygdala (ACo) and periamygdaloid cortex (PAC) (Carmichael et al., 1994).

Whether the human olfactory system mirrors the OB-amygdala projections found in animals remains poorly understood, though there is some evidence to consider. Two early post-mortem histology studies conducted on a small number of subjects (Allison, 1954;Crosby & Humphrey, 1941) suggested that the human OB projects to the MeA, ACo, and PAC. Allison implied that the OB likely also projects to the central nucleus (CeA) in humans, but was unable to confirm this (see pp 485). Based on this early work, human studies have assumed that MeA, ACo, and PAC comprise human “primary olfactory amygdala” (Noto et al., 2021). However, this classification has not been systematically tested in a larger group of subjects.

Our lack of understanding of the connections between the olfactory bulb and amygdala in humans has been due, in part, to technical limitations. The olfactory bulb is small, and located near areas of high magnetic inhomogeneity, which distorts magnetic resonance imaging (MRI) signals. Furthermore, understanding the detailed anatomy of connections between the OB and amygdala requires the ability to measure discrete amygdala subregions, which are small and difficult to define using MRI. Tracing studies in humans are technically difficult and require post-mortem techniques which carry limitations. However, recent advancements in diffusion-weighted MRI (dMRI) have made it possible to study the lateral olfactory tract in humans (Echevarria-Cooper et al., 2022;Fjældstad et al., 2017;Milardi et al., 2017;Skorpil et al., 2011), and the structural properties of amygdala subregions (Bach et al., 2011;Ball et al., 2007;Bzdok et al., 2013;Entis et al., 2012).

Here, we applied state-of-the-art dMRI techniques, using an acquisition protocol that was optimized for imaging olfactory areas (Echevarria-Cooper et al., 2022), to study the*in vivo*connections between the olfactory bulb and the amygdala in 25 healthy human participants. We first aimed to test the hypothesis that the olfactory bulb exhibits a higher density of white matter tracts connecting to ACo, MeA, and PAC than other amygdala subregions. We then aimed to characterize the anatomical connectivity between the ACo, MeA, and PAC and the rest of the brain.

## 2 Methods

### 2.1 Dataset

Diffusion, T1- and T2-weighted MRI data were collected between 2018 and 2019 for an earlier study, which is described in detail elsewhere (Echevarria-Cooper et al., 2022). Here, we briefly summarize relevant details of the dataset.

### 2.2 Participants

Data include scans from 27 participants (14 male and 13 female; age: mean 25.76 ± SD 4.01). Two participants, both males, were excluded from final analyses because they did not complete the MRI scanning protocol. All participants were right-handed with no neurologic disorders, psychiatric disorders, or MRI contraindications. This study was approved by the Institutional Review Board at Northwestern University under protocol number STU00098371, and the study adhered to the Declaration of Helsinki and the Belmont Report. Participation was voluntary, and written informed consent was obtained from all participants.

### 2.3 Olfactory perceptual testing

Participants underwent olfactory perceptual testing using the Sniffin’ Sticks threshold (n-butanol), discrimination, and identification tests administered in the listed order. Subsequently, participants repeated the olfactory threshold test, and the two threshold scores were averaged. All participants scored above anosmic thresholds for all three tests; 8 participants scored in the hyposmic range, all of whom scored close to the lower end of normosmia. To ensure that these participants did not skew the results, we ran several analyses looking for any relationships between olfactory function and properties of LOT to amygdala structural connectivity. We did not find any significant relationships (Supp. Fig. 4).

### 2.4 Head stabilization

All participants wore personalized custom-made head stabilizers during the MRI scanning sessions in order to minimize head movement (Power et al., 2019). Head stabilizers were created using 3D images of each participant’s face captured by a hand-held camera and the Caseforge IOS app. The resulting file was used to mill the stabilizer from rigid foam to fit each participant’s face on the inside, and the MRI head coil on the outside.

### 2.5 MRI data acquisition

The MRI scanning was performed on a 3T Siemens Prisma scanner with a 64-channel head-neck coil. For each participant, we collected a set of diffusion-weighted images, a T1-weighted image, and a T2-weighted image. To collect the dMRI images, we used the RESOLVE dMRI scanning protocol (Porter & Heidemann, 2009) with seven readout segments. This sequence uses multiple RF pulses to re-excite the tissue and collect the data in multiple segments in the readout direction, with segments combined in the end to create the final image. This allows for a shorter TE and reduced susceptibility artifacts. We also used a navigator echo to monitor between-segment motion, and re-acquired volumes that suffered from excessive motion (Porter & Heidemann, 2009). Simultaneous multi-slice acquisition (Nunes et al., 2006) was used to increase the spatial coverage of scans. Combined, these methods largely eliminated susceptibility artifacts.

Diffusion-weighted scans were acquired at an oblique slice angle (~30° relative to the AC–PC plane) to further reduce susceptibility artifacts (Weiskopf et al., 2006). Detailed scanning parameters were as follows: 1.5 mm isotropic voxels, 92 slices; field of view (FoV) = 240 mm; matrix size = 240 × 240 × 138 mm; 90 diffusion-weighted directions at b = 1000 s/mm^2^; 12 interspersed b0 volumes; phase encoding = A > P; TE1 (image echo) = 61 ms; TE2 (navigator echo) = 98 ms; repetition time (TR) = 6250 ms; flip angle = 180°; and bandwidth = 897 Hz/Px, multiband factor = 2.

T1-weighted structural scans were acquired at a resolution of 1.0 mm isotropic, TE = 2.94 ms, TR = 2300 ms, flip angle = 9°, FoV = 256 mm, matrix size = 256 × 256 × 176 mm; phase encoding = A > P, bandwidth = 240 Hz/Px.

The T2-weighted structural scans covered the ventral frontal lobes and temporal poles, including the olfactory bulbs, orbitofrontal cortex, lengths of the olfactory tracts, and amygdala. These scans were acquired with the Siemens ZOOMit protocol, at a resolution of 0.5 mm isotropic, TE = 125 ms, TR = 1000 ms, flip angle = 100°, FoV = 160 mm, matrix size = 82 × 160 × 72 mm, phase encoding = A > P, and bandwidth = 256 Hz/Px.

### 2.6 MRI data processing

All MRI data were converted to the NiFTI file type using MRIcron’s*dcm2niix*function (Li et al., 2016). The diffusion MRI data were corrected for motion and eddy current artifacts using FSL’s*eddy_openmp*function (Jenkinson et al., 2012;Smith et al., 2004;Woolrich et al., 2009). The T1- and T2-weighted images were co-registered to the native diffusion space using the SPM12 toolbox in MATLAB. Diffusion image processing and analysis were performed with the MRtrix3 toolbox. We estimated the response function for white matter using*dwi2response*for single tissue type (Tournier et al., 2007), and fiber orientation distribution was generated using the*dwi2fod*function. All tractographies were generated using the*tckgen*function. Detailed inclusion and exclusion settings and parameters regarding each analysis are described in the corresponding results inSection 3. Streamlines were mapped to streamline density maps using*tckmap*with the original*-tdi*option, in which each streamline contributes a value of unity for each voxel in the final map. Streamline endpoint maps were constructed using the*tck2connectome*function with the*-vector*option, so that only the endpoint distribution in our target (and not the seeding) ROIs was included in our analysis. For the group-level streamline endpoint analysis, we obtained the MNI coordinate for each projection endpoint and plotted all endpoints across participants on a 1 mm MNI brain.

### 2.7 Statistical analysis

All statistical analyses were conducted using custom-made MATLAB (RRID: SCR_001622) scripts. For distribution fitting, we used*poissfit*to estimate the parameter lambda, which in our case represents the mean number of streamlines per voxel, for the region that had the least number of streamline endpoints. This number was used to generate a random Poisson distribution using*poissrnd*. This simulated Poisson distribution functioned as a null distribution describing the number of endpoints per voxel that would be observed due to noise. The threshold for statistical significance was set to p = 0.05, representing 1 endpoint per voxel. In other words, if a given voxel had more than 1 endpoint, it was statistically unlikely (p < 0.05) that the connection we observed was stemming from noise. For k-means clustering, we used the*kmeans*function to cluster the subregions based on the proportion of significantly connected voxels. The initiation for each clustering was random, and we used squared Euclidean distance as the distance measurement in clustering. The number of clusters was always set to 2. We repeated the process 1000 times to calculate the probability that each of the regions was clustered with PCo (seeSection 3for details). To provide statistics for the clustering probability, we generated a sham condition in which we sampled the same number of data points randomly across all subregions, and calculated the probability with which this sham condition was clustered with PCo. This random sampling was permuted 1000 times to generate a null distribution for the probability of clustering with PCo. The threshold for statistical significance was set at p = 0.05. We computed the Dice coefficient (Dice, 1945) between endpoint density maps and each amygdala subregion ROI with the MATLAB function*dice.*Specifically, binarized volumetric images for each ROI and projection endpoint density map were collapsed into a 2d image along the third dimension when read into MATLAB using FSL’s*MRIread*function, and binarized to calculate the Dice coefficient.

### 2.8 Streamline density value analysis

Streamline density maps for each participant were projected into the 1 mm MNI space for group-level analyses. For whole-brain connectivity pattern analysis, we defined targets using the regions in the Harvard-Oxford Cortical and Subcortical Brain Atlas included in the FSL toolbox. Streamline density maps for each participant in 1 mm MNI space and the corresponding atlas were read into MATLAB; then, the sum of streamline density values across all voxels for each region was calculated. The sum of streamline density values was normalized to the number of seeding attempts. Statistics for streamline density value comparisons were generated across participants.

### 2.9 ROIs

ROIs for the lateral olfactory tract were generated in a previous publication (Echevarria-Cooper et al., 2022), and are available online (Left LOT:https://neurovault.org/images/511896/; Right LOT:https://neurovault.org/images/511897/). We hand-drew the ROIs for the optic nerve and the arachnoid cistern on each participants’ native space and used them as exclusion masks to avoid streamlines entering these areas. We hand-drew all amygdala subregion ROIs on a standard MNI brain using the Atlas of the Human Brain (Mai et al., 2015) and previously published literature (Noto et al., 2021) as references. All ROIs are freely available online athttps://neurovault.org/collections/20559/. Because human olfactory amygdala subregions are not well-studied, and may be unfamiliar to some, we provide here a detailed description of how we drew these ROIs on a standard brain. In detail, to outline the MeA and ACo, we first identified their anterior limits in the coronal plane. The anterior limit of MeA coincides with the posterior edge of temporal piriform cortex (Mai et al., 2015), typically around MNI coordinate y = -4. We followed published methods to identify the posterior limit of piriform cortex (Zelano et al., 2007), and then marked the anterior edge of MeA on the next coronal slice, 1 mm in the posterior direction from the posterior edge of piriform cortex. Around this coronal slice, the surface of the entorhinal sulcus changes from flat and smooth when contiguous with piriform cortex, to angled and exhibiting a distinct small bump, which indicates the position of the ACo. This landmark is visible in conventional individual T1 scans. The posterior borders of the MeA and ACo were identified using the optic nerve as a landmark. Specifically, these regions continue in the posterior direction until the optic nerve nearly touches the amygdala surface, and the anterior border of the hippocampus becomes visible. The anterior border of the PAC starts when the temporal and frontal lobes fuse on the coronal slice, and extends to the anterior edge of the temporal piriform cortex according to Mai et al. atlas. These ROIs were hand-drawn by author Q.Y., and verified by author C.Z.

## 3 Results

### 3.1 The MeA, ACo, PAC, and CeA have denser connections with the olfactory bulb

**Fig. 1.**
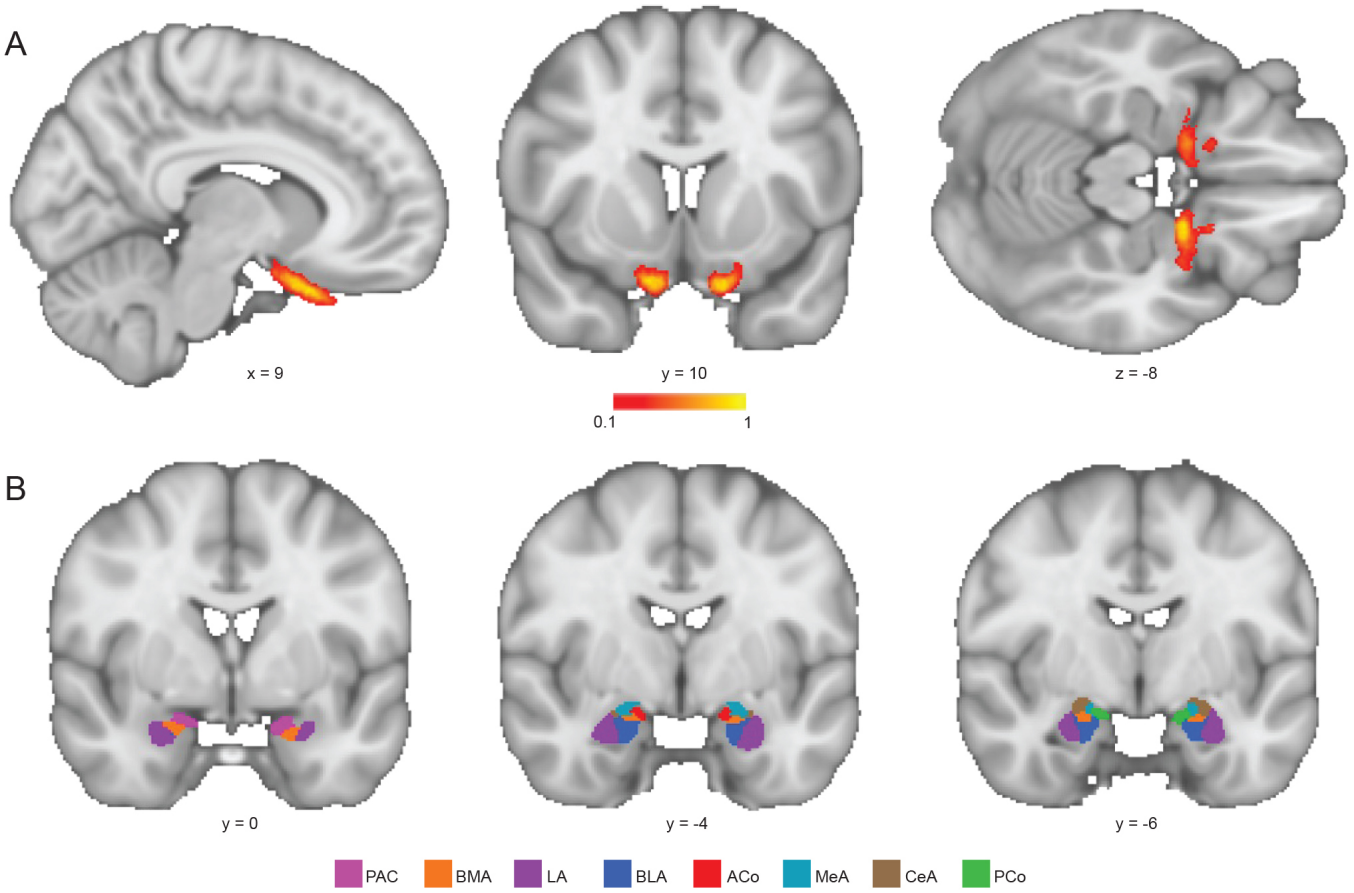
Seeding and target regions used in streamline analyses. (A) Atlas of the lateral olfactory tract, taken from (Echevarria-Cooper et al., 2022). (B) Amygdala subregion ROIs based onMai et al. (2015).

To determine which subregions of the amygdala have denser connections with the OB in vivo, we conducted streamline analyses for each amygdala subregion, including MeA, CeA, ACo, PAC, LA, BLA, BMA, and PCo (Fig. 1B). As discussed in prior work (Echevarria-Cooper et al., 2022), direct streamline connections seeding from the OB to the rest of the olfactory cortical areas are nearly impossible to generate due to the susceptibility artifact induced by sinuses nearby. This creates a gap in the signal which prevents continuous tracking. We therefore, used the lateral olfactory tract (LOT) atlas generated in the previous publication (Echevarria-Cooper et al., 2022) as the seeding point (Fig. 1A), as it contains the white matter through which the olfactory bulb projects to the rest of the human brain. For the streamline endpoint analysis, we first generated streamlines seeding from the ipsilateral LOT to the entire amygdala. We excluded the optic nerve, the arachnoid cistern, and major grey matter structures nearby, including the ACC, the OFC, and the insula as controls. We then characterized the streamlines by mapping the location of all endpoints in the amygdala in a voxel-by-voxel manner in each participant, then projected this endpoint map into MNI space to examine the distribution of endpoints across different subregions and participants.

**Fig. 2.**
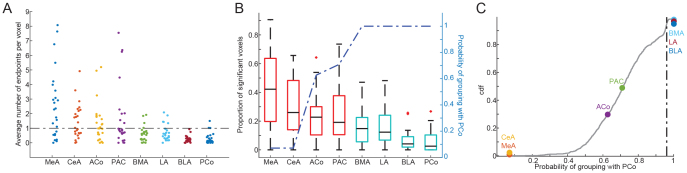
Clustering of amygdala subregions based on structural connectivity with the OB. (A) Scatter plot showing number of endpoints averaged across voxels for each subregion in each subject. Dashed-black line indicates threshold for statistical significance compared to chance level. (B) Box plot showing proportion of significant voxels for each subregion (y-axis on left). Dashed-blue line indicating probability at which a given subregion is clustered with PCo by a randomly initiated k-means clustering algorithm (y-axis on right). (C) Cumulative distribution function (CDF) of permutation distribution for the k-means cluttering process. Grey line indicates CDF of permuted sham conditions being grouped with PCo. Colored dots represent real probability for each subregion. Black-dashed line indicates p = 0.05 threshold.

**Fig. 3.**
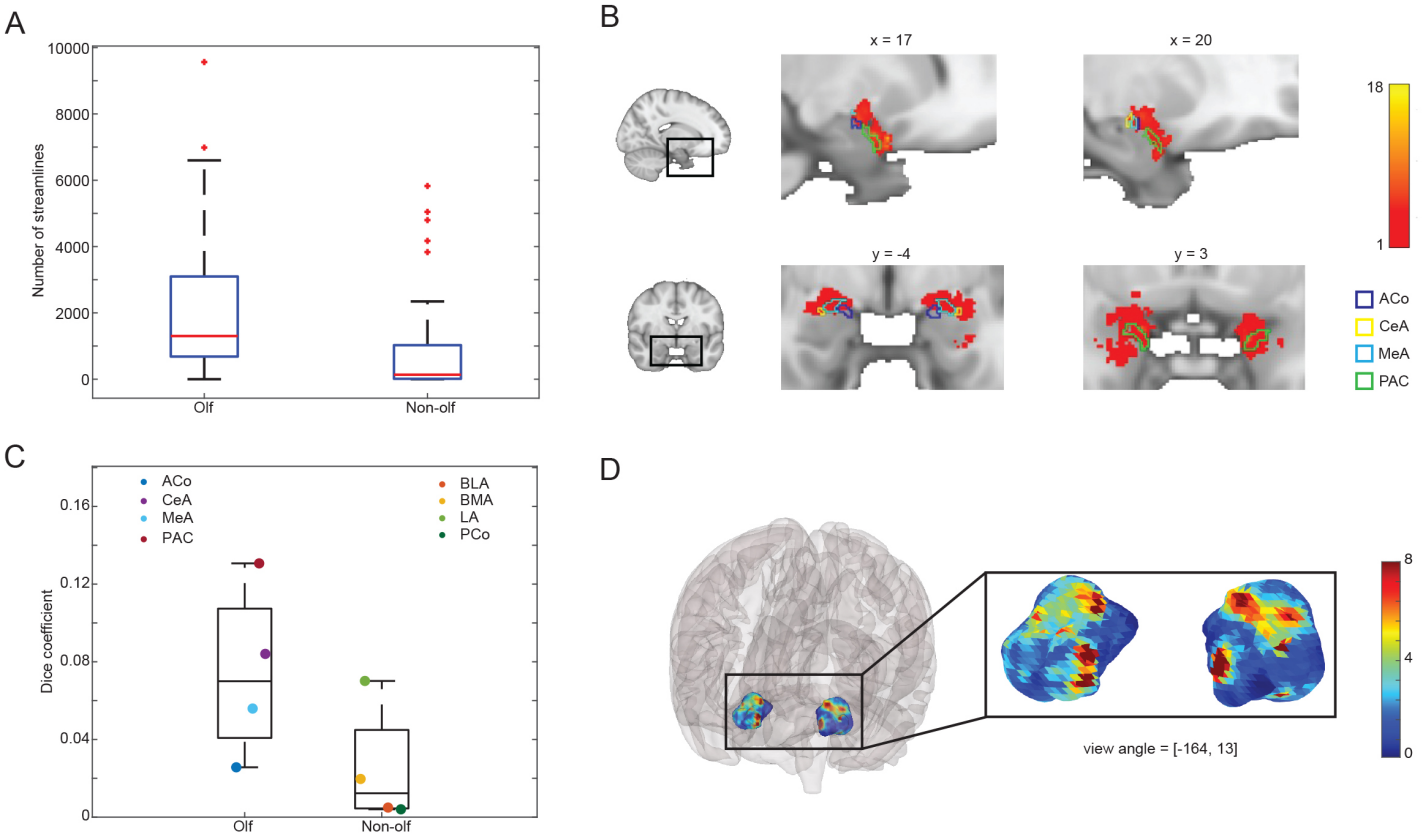
Distribution of OB to amygdala structural connectivity. (A) Number of unique streamline connections from LOT to ipsilateral olfactory vs. non-olfactory amygdala subregions. Red line indicates median. Box limit indicates the 25th and 75th percentile. Whiskers indicate the upper and lower limit of data excluding outliers (+/-2.7 standard deviations). Outliers are plotted as red crosses. Olfactory amygdala subregions show significantly higher connectivity (paired t-test, p = 2.0284e-05). (B) Colocalization of LOT-to-amygdala streamline endpoints and olfactory amygdala subregions. Colored contours outline the amygdala subregions. Colormap indicates number of endpoints in each voxel averaged across participants. Black boxes on the whole-brain slice image indicate the position of zoomed-in images relative to the whole brain. (C) Dice coefficient for projection endpoint map in panel B and each amygdala subregion. (D) Heatmap of LOT-to-amygdala streamline endpoint density on an inflated 3D amygdala surface (with and without glass brain). Color indicates the number of streamline endpoints for each vertex, averaged across participants.

Connections with the LOT were not uniform across amygdala subregions (Fig. 2A; one-way ANOVA across amygdala subregions: Df = 7, F = 9.3057, p = 6.5574e-10). Subregions ranked according to the number of endpoints per voxel from most to least: MeA, CeA, ACo, PAC, LA, BLA, BMA, and PCo (Fig. 2A). We next statistically compared these values in order to divide amygdala subregions into two groups: those that likely receive direct input from the OB (olfactory subregions), and those that are unlikely to receive direct input from the OB (non-olfactory subregions). In order to generate an unbiased grouping of these regions, we first needed to estimate a threshold for chance-level endpoint numbers per voxel. To do this, we reasoned that the amygdala subregion with the fewest endpoints would serve as an approximation of chance connectivity. Specifically, as some amygdala subregions do not receive input from the olfactory bulb (Allison, 1953,^1954^;Carmichael et al., 1994), any endpoints detected in the subregion with the fewest number of endpoints in our data should have been due to random noise embedded in the imaging data and streamline fitting process. In our data, that subregion was the PCo, which has previously been reported not to receive fibers from the human OB (Allison, 1954). Thus, we fitted a Poisson distribution of endpoint numbers per voxel and estimated the mean rate of connectivity of the PCo. We then used this mean connectivity value to generate a null distribution of endpoint densities for a given voxel at chance level, and compared all voxels against this null distribution to estimate which voxels were likely connected with the LOT and which were not. For each subregion, we calculated the proportion of voxels with a p-value of less than 0.05 when compared against this null distribution (Fig. 2B), and used these results to produce unbiased clustering of the regions into two groups. We randomly initiated 1000 k-means clustering processes on the data in order to compute the probability that each subregion was clustered with PCo. We found that across all iterations, the PCo, BLA, LA, and BMA always clustered as a single group, with 100% probability (Fig. 2B, blue dashed line), suggesting that these four subregions are unlikely to connect with the LOT. Permutation testing confirmed that the probability of this grouping was statistically significant (p < 0.05, permutation test), whereas none of the other regions (Mea, CeA, ACo, and PAC) were significantly co-clustered with PCo (seeSection 2). A control analysis using the distance between each subregion and the center of LOT as the measurement for clustering yielded different groupings (seeSupp. Fig. 3), suggesting that these findings were not driven by distance.

Based on these results, we conclude that MeA, CeA, ACo, and PAC are likely to form connections with the LOT, whereas the BMA, LA, BLA, and PCo are not. Therefore, we suggest that the MeA, CeA, ACo, and PAC constitute the “olfactory subregions” of the amygdala, and BMA, LA, BLA, and PCo constitute the “non-olfactory subregions”. This fits with early anatomical findings (Allison, 1954), and provides additional evidence that the CeA might receive direct input from the OB in humans.

In line with our data-driven classification of amygdala subregions into olfactory and non-olfactory counterparts, a combined analysis comparing the two groupings revealed stronger connectivity between the LOT and olfactory subregions (Pooled t-test between two groups inFig. 2A: t = 7.3068, df = 99, p = 7.0936e-11). In a separate confirmatory analysis, we compared the number of unique streamline connections between the LOT and the ipsilateral amygdala subregions. For each subject, we generated streamline connections using LOT as the seed region, and ipsilateral olfactory or non-olfactory amygdala subregions as the target, while excluding the other amygdala subregions, the optic nerve, the arachnoid cistern, and major grey matter structures nearby, including the ACC, the OFC, and the insula. After 100,000 seeding attempts, we counted the number of streamlines generated for each subject from the LOT to each amygdala subregion. We found that across subjects, the olfactory amygdala subregions received significantly more unique connections than the non-olfactory subregions (Fig. 3A; Paired sample t-test. t = 3.7032, df = 49 (left and right counted separately) p = 5.4027e-04). To further demonstrate the co-localization of streamline connection endpoints and the olfactory amygdala subregions, we plotted the endpoint distribution map in MNI space using a threshold of p = 0.05 in the distribution of chance-level connections (Fig. 3B). To quantify the overlap, we calculated the Dice coefficient between the map and each amygdala subregion (Fig. 3C). We found that olfactory amygdala subregions exhibited a consistently higher Dice coefficient than non-olfactory subregions, except for LA. It is worth noting that the Dice coefficient describes only the extent of overlap of two binary images, and therefore the strength of connection that overlaps with each amygdala subregion is better captured inFigure 2. Together, these results validated our data-driven definition of olfactory and non-olfactory subregions, and indicated that MeA, CeA, ACo, and PAC form significantly denser connections with the OB than BLA, BMA, LA, and PCo.

### 3.2 The olfactory and non-olfactory subregions of the amygdala form distinct structural connectivity patterns with the rest of the brain

**Fig. 4.**
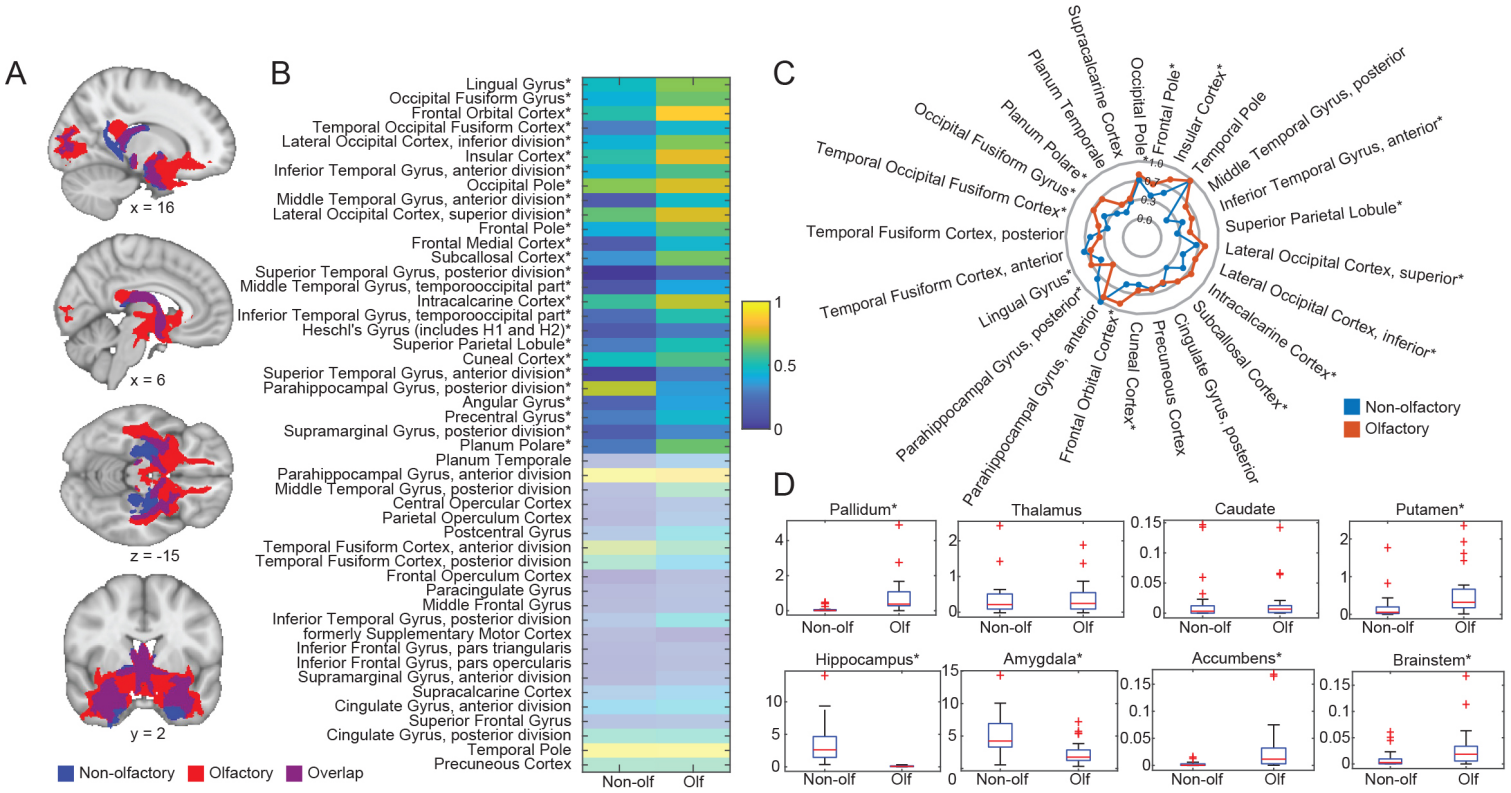
Structural connectivity of olfactory vs. non-olfactory amygdala subregions. (A) Streamline density map for non-olfactory only (blue), olfactory only (red), and overlap (purple). Maps are thresholded at 1 streamline per participant and binarized. (B) Streamline density value from olfactory vs. non-olfactory subregions to 48 cortical targets from Harvard-Oxford Cortical Brain Atlas (averaged across participants, normalized from 0 to 1 for plotting). Asterisk indicates significant differences between amygdala subregion groups (paired t-test, FDR-corrected p < 0.05. Statistical test performed on original data.). Non-significant areas in the heatmap are shaded. (C) Radar plot showing all connections that were present in more than 50% of the participants between cortical regions and olfactory vs. non-olfactory amygdala subregions. Connection strength is normalized from 0 to 1 for plotting. Connections with olfactory amygdala shown in red and non-olfactory in blue. (D) Boxplot showing streamline density values between olfactory vs. non-olfactory subregions and eight subcortical regions from Harvard-Oxford Subcortical Brain Atlas (excluding neocortex and ventricles). Asterisk indicates statistically significant difference between amygdala subregion groups (paired t-test. FDR corrected p < 0.05).

We next examined whether the olfactory and non-olfactory amygdala, as groups, differ in their structural connectivity patterns with the rest of the brain. To do this, we created two ROIs—an olfactory amygdala ROI comprising MeA, ACo, and PAC, and a non-olfactory amygdala ROI comprising BLA, LA, BMA, and PCo. We then performed free-tracking seeding from each group, and analyzed their resulting streamline density maps to the whole brain. Because CeA is the major output nucleus of the amygdala (Fadok et al., 2018) and would therefore introduce connections from non-olfactory subregions, we used it as an exclusion mask in this analysis (though seeSupp. Figs. 1 and 2for results with CeA left in). An equal number of streamlines (n = 100,000) were generated for each tracking attempt, and the streamlines were converted to a density map which was then registered to MNI space and mapped to the Harvard-Oxford Human Cortical and Subcortical Brain Atlas (Desikan et al., 2006;Frazier et al., 2005;Goldstein et al., 2007;Makris et al., 2006). In order to examine whether olfactory and non-olfactory amygdala subregions form distinct whole-brain structural connections, we first thresholded the two streamline density maps at 1 streamline per participant and binarized the maps to examine their unique vs. shared connectivity. We found that the olfactory and non-olfactory regions of the amygdala form different structural connectivity patterns with the rest of the brain (Fig. 4A). Next, we examined whether the subregion groups connect with whole-brain targets with different strengths, including cortical and subcortical areas. We found that 26 of the 48 cortical regions defined by the atlas formed connections that statistically differed in strength across the two groups (Fig. 4B) (Wilcoxon signed-rank test across participants, FDR corrected p < 0.05). To show the most prominent structural connectivity partners of olfactory vs. non-olfactory amygdala groups, we created a radar plot showing the streamline connections that were present in at least 50% of the participants (Fig. 4C). These analyses of cortical connectivity found that the olfactory subregions showed more streamline connections with multiple regions of the temporal lobe as well as the OFC and insula. In contrast, the non-olfactory subregions showed more streamline connections with fusiform and parahippocampal areas. We conducted the same analysis for subcortical regions, and found that the olfactory subregions showed more streamline connections with the pallidum, the putamen, the accumbens, and the brainstem, whereas the non-olfactory subregions showed more streamline connections within amygdala, as well as with the hippocampus (Fig. 4C) (asterisk indicates FDR corrected p < 0.05, Wilcoxon signed-rank test). We then mapped these results on a standard brain (Fig. 4D): the areas forming statistically stronger connections with the olfactory amygdala are shown in red, and the areas forming stronger connections with the non-olfactory subregions are shown in blue.

### 3.3 Each olfactory amygdala subregion forms distinct structural connections with the rest of the brain

**Fig. 5.**
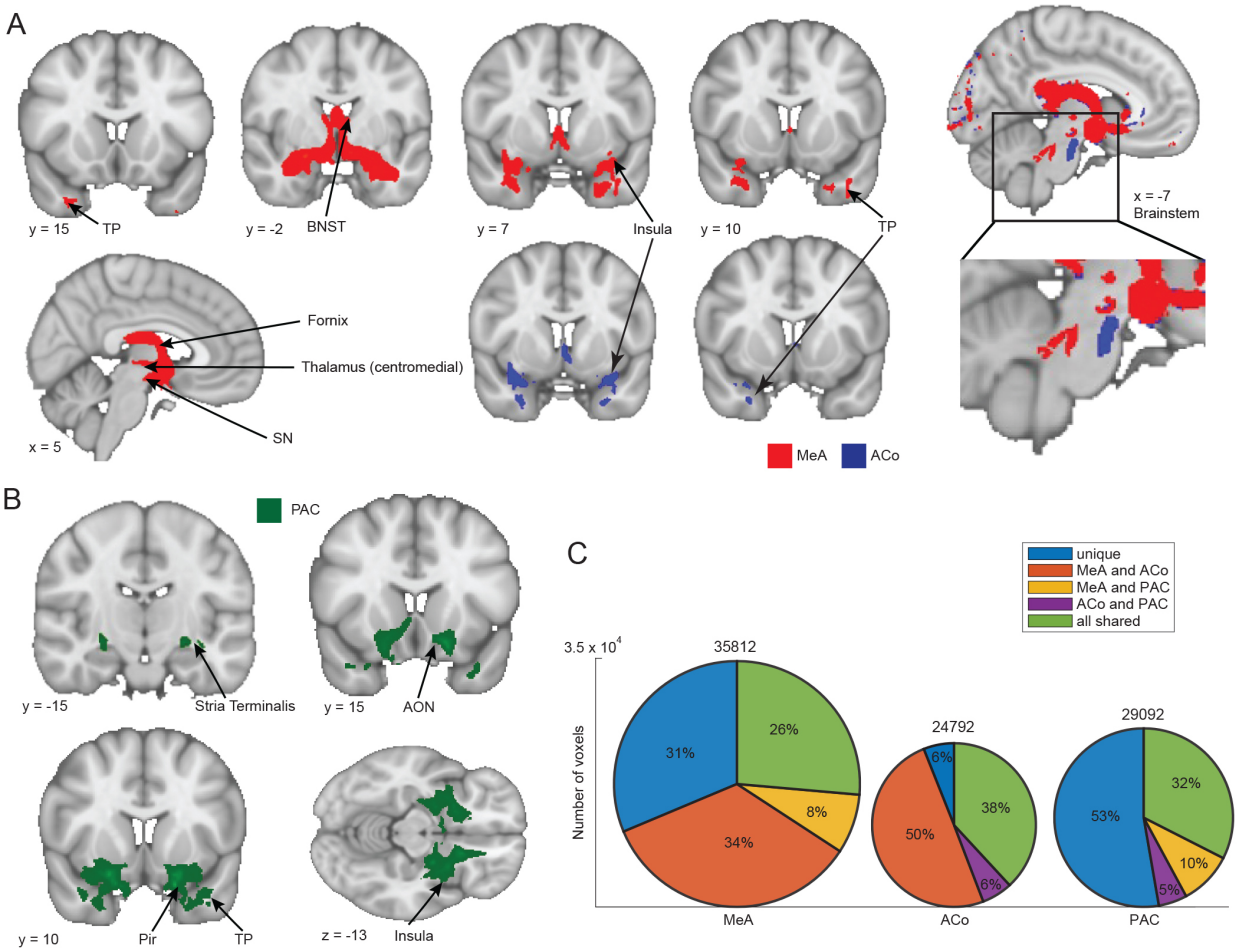
Streamline density map of free-tracking seeding from each olfactory amygdala subregion, thresholded to the upper 5th percentile of all non-zero track density values. (A) Streamline density map of MeA and ACo. (B) Streamline density map of PAC. (C) Pie chart showing percentage of unique vs. shared voxels in streamline density maps among the olfactory amygdala subregions. Number above each pie chart indicates the total number of non-zero voxels in each thresholded streamline density map. Size of the pie chart is proportional to the number of non-zero voxels in the streamline density map for each olfactory amygdala subregion. TP: temporal pole, BNST: bed nucleus of stria terminalis, SN: substantia nigra, AON: anterior olfactory nucleus, Pir: piriform cortex.

We next performed a detailed analysis of the structural connectivity of each olfactory amygdala subregion. For each participant, we performed free-tracking from MeA, ACo and PAC with equal number of streamlines (n = 5,000), and converted the result to a streamline density map. The maps for each individual were projected into MNI space and averaged across all participants. We thresholded the group-averaged streamline density map at the upper 5th percentile of the non-zero values for each map, and binarized the maps for further visualization and analysis (Fig. 5Aand5B). We next counted the number of non-zero voxels in each of the thresholded streamline density maps, and grouped voxels based on their uniqueness (Fig. 5C.). This provided a description of how unique the structural connectivity patterns are for each olfactory amygdala subregion. Overall, we found that the MeA had the highest number of streamline connection voxels, and ACo had the lowest (Fig. 5C). These results agree with previous work on whole-brain functional connectivity of these amygdala subregions, showing that among these amygdala subregions, the MeA forms maximal functional connectivity patterns with the rest of the brain, and the ACo forms the least (Noto et al., 2021).

Notable structural connectivity partners of MeA included the temporal pole, perirhinal cortex, insula, BNST, and parts of the thalamus and SN. We found that most of the structural connectivity partners for ACo were shared with MeA. They generally connected with the same cortical areas, though sometimes in a non-overlapping manner, such as in the insula and perirhinal cortex (Fig. 5A). It is worth noting that when examining the connectivity map with a looser threshold, the MeA and CoA also connected with parts of the brainstem in a non-overlapping manner (Fig. 5A). Specifically, the MeA potentially connects with the parabrachial nuclei, while the ACo potentially connects with the pontine nucleus (Rushmore et al., 2020). This suggests the possibility that these olfactory amygdala subregions could play differential roles in aspects of breathing control.

In contrast with the MeA and ACo, the PAC exhibits more olfactory-related connections, including the piriform cortex, AON, and insula.

## 4 Discussion

Historically, scientific investigations of the human amygdala have largely considered it as a single region without differentiating between subregions, due mainly to technical limitations. However, the amygdala comprises cytoarchitecturally distinct subregions, which subserve different sensory and cognitive functions (Janak & Tye, 2015;LeDoux, 2007) and the differences in connectivity of these subregions is poorly understood in olfaction. In rodents, a subset of amygdala subregions receive direct, single-synapse projections from the olfactory bulb, including the MeA, ACo, PCo, and PAC. Whether such architecture is conserved in humans has been an open question, with very limited existing supporting evidence (Allison, 1954). Here, we used dMRI optimized for olfactory areas in a group of 25 healthy human participants to systematically quantify anatomical connections between the olfactory bulb (using the olfactory tract as a proxy) and all amygdala subregions. We found that the human OB connects with MeA, ACo, PAC, and CeA, but not with PCo, BLA, LA, and BMA. We used convergent data analysis techniques to group human amygdala subregions into non-olfactory and olfactory counterparts. An initial data-driven k-means approach was based on strength of connectivity with the OB. This revealed two clusters: a cluster comprising PCo, BLA, LA, and BMA, which were grouped, consistently across permutations, as a single cluster exhibiting low or no connectivity with the OB; and a cluster comprising MeA, ACo, PAC, and CeA, exhibiting higher connectivity with the OB. Confirmatory analyses demonstrated both stronger and denser connectivity between regions in the second group and the OB than those in the first group. We therefore propose that MeA, ACo, PAC, and CeA are the olfactory subregions of the amygdala.

Among these olfactory amygdala subregions, the CeA is distinct. It is the major output nucleus of the amygdala and is known to have non-olfactory functions (Fadok et al., 2018). Though it has been suggested that CeA could potentially receive monosynaptic projections from the OB in humans, evidence is thin—based on a single study of two subjects, one of which was pathological (Allison, 1954). Here, our data suggest that CeA exhibits strong anatomical connectivity with the OB. Although we categorized CeA as “olfactory amygdala” based on our data-driven approach, the fact that CeA receives input from all amygdala subregions and functions as a major output relay argued against including CeA in whole-brain structural connectivity analyses (i.e.Figs. 3and4). Doing so would likely reduce the olfactory specificity of our findings, due to structural connectivity between CeA and other amygdala subregions, and thus other sensory modalities. Therefore, we did not include CeA in these subsequent analyses. Further anatomical verification is needed to confirm consideration of CeA as part of the primary olfactory cortex, and to examine its specific olfactory functions.

We subsequently characterized structural connectivity between olfactory and non-olfactory subregions with the rest of the brain. We found that the olfactory and non-olfactory subregions of the amygdala showed different structural connectivity patterns in relation to cortical and subcortical targets. Specifically, the olfactory subregions formed stronger connections with multiple regions of the temporal lobe, the OFC, and the insula, as well as pallidum, the putamen, the accumbens, and the brainstem. In contrast, the non-olfactory subregions connected more strongly with fusiform and parahippocampal areas. Intriguingly, we found that the connections with the majority of the cortical targets defined by the Harvard-Oxford Cortical Atlas were stronger from the olfactory subregions of the amygdala compared to the non-olfactory subregions. This is in line with recent work suggesting that olfaction has unique early cortical access (Menelaou et al., 2024), and therefore primary olfactory areas may project—via dual pathways—to multiple cortical areas, supporting the concept of an olfactocentric cortical system that developed during brain evolution (Waymel et al., 2020). That said, several factors in our analyses could contribute to this result. Most notably, the MeA is a highly multisensory area with broad connections across cortex, subcoritcal areas and midbrain. Though it is defined as an olfactory subregion of the amygdala based on its connection with the OB, this designation is not meant to be exclusive: that is, the MeA’s function is not related to olfaction alone. And therefore, our results do not imply that the structural connections formed by olfactory amygdala subregions serve the olfactory system alone. For example, reflecting these multisensory structural connections, a functional imaging study found that response selectivity to human faces is specific to the superficial nuclei of the amygdala, which overlap substantially with the olfactory subregions defined in our study (Goossens et al., 2009). Due to limitations inherent to dMRI methods (discussed below), detailed circuit mapping experiments are needed to fully characterize their functions.

We then characterized the structural connectivity of each olfactory amygdala subregion with the rest of the brain, and found that overall, MeA had the largest number of unique connections compared to the ACo and PAC. We also found that whole-brain structural connectivity was heavily overlapping between MeA and ACo, whereas the connectivity map of PAC was more distinct. This is in line with prior work on the functional connectivity of these amygdala subregions (Noto et al., 2021).

MeA is a particularly interesting olfactory brain area in humans. Its rodent counterpart is often referred to as “accessory olfactory cortex”, because it receives dense projections from the accessory olfactory bulb, which humans lack entirely. Why do humans have an MeA, and what is it doing in an animal with no accessory olfactory system? Very little is known about this region in humans, yet it constitutes an intriguing example of divergence in a highly conserved system. In rodents, the MeA is involved in odor-guided social behaviors, including mating (Hadeishi & Wood, 1996;Takenawa et al., 2023;Wood & Newman, 1995), defensive behaviors (Miller et al., 2019), and aggression (Nordman et al., 2020;Padilla et al., 2016;Zhang, 2021). Many of these behaviors are mediated by connections between the MeA and the BNST. In line with this, our data showed connectivity between MeA and BNST in humans. Broadly speaking, our findings show that human MeA is structurally connected with a number of brain areas involved in social behavior, including the temporal pole (Dipani et al., 2024;Pehrs et al., 2017) and substantia nigra (Batten et al., 2024;He et al., 2021). This raises the possibility that in humans, MeA may still be involved in odor-guided social behaviors despite the lack of an accessory olfactory system. That said, primate work has shown that the MeA is a multisensory region, and is involved in non-olfactory social behaviors as well (Gothard, 2020), and this is likely the case in humans (see, for example,Edmonds et al., 2024;Noto et al., 2021). Overall, our findings support the possibility of conserved functionality of MeA across species despite dramatically different sensory input patterns.

In addition to connections with brain areas involved in social behaviors, we also found that the MeA, along with the ACo, connects with brainstem nuclei involved in respiratory control, including the pontine and parabrachial nuclei (Chamberlin, 2004;Dutschmann & Dick, 2012;Zuperku et al., 2017). This finding dovetails intriguingly with work in the field of epilepsy showing that the amygdala is implicated in breathing disruption, including apnea (Nobis et al., 2018;Rhone et al., 2020), and is therefore involved in respiratory control. Intriguingly, the “amygdala inhibition of respiration site” (AIR) identified byRhone et al. (2020)was largely made up of the cortical and medial amygdala subregions, which overlap with olfactory amygdala subregions. Our findings that these olfactory amygdala subregions connect with parts of the brainstem that modulate breathing adds to the evidence that they are involved in the pathology of seizure-induced apnea, which may be of profound clinical importance in Sudden Unexpected Death in Epilepsy (SUDEP), the leading cause of death in epilepsy patients.

Breathing modulation also plays a critical role in odor sampling, through sniffing (Mainland & Sobel, 2006). Sniffing patterns change rapidly and unconsciously during odor sampling, suggesting that the olfactory system contains within it a circuit that can override and modulate respiration. Speculatively, since MeA and ACo receive direct projections from the olfactory bulb and are likely involved in respiratory control, they may also be involved in rapid sniffing modulation induced by olfactory stimuli in humans (Johnson et al., 2003). The olfactory amygdala is a good candidate for this unknown olfactory circuit, the understanding of which is important to elucidating human odor coding.

Our data show that PAC has more specialized connections to areas related to olfactory cognitive processing than the MeA or ACo, and therefore may possess a more specialized functional role in olfactory processing. Previous work in rodents has shown that the PAC connects heavily with other amygdala subregions, as well as olfactory areas such as the piriform cortex (Majak et al., 2004). However, little is known about its function; in contrast to MeA, the PAC is less studied in rodents, and a consistent name for this region has not emerged. The PAC is variously referred to as periamygdaloid cortex (McDonald, 1998), anterior amygdala area (Rhone et al., 2020), and amygdalo-piriform transition area (Jolkkonen et al., 2001). Some consider it part of the piriform cortex (Paxinos & Watson, 2006). There have been few systematic experiments probing its function. Early anatomical studies suggested the PAC receives main olfactory bulb projections, but not accessory olfactory bulb projections (McDonald, 1998). Our results here support conservation of OB connectivity with PAC in humans, with evidence for PAC being more specialized in olfactory perception than other olfactory amygdala subregions.

Advances in imaging technology have increasingly allowed consideration of distinct subregions of the amygdala. While separate consideration of the superficial, centromedial and basolateral parts of the amygdala has appeared in multiple prior human diffusion neuroimaging studies, few have looked specifically at the relationship between the amygdala subregions and the olfactory system (Bzdok et al., 2013). Some dMRI-based parcellation studies have divided the human amygdala into the several parts based on whole-brain structural connectivity and direction of primary diffusion (Bach et al., 2011;Rizzo et al., 2018;Saygin et al., 2011;Solano-Castiella et al., 2010). Our study adds to the available evidence that the medial-cortical portion of the human amygdala is both structurally and functionally distinct from the basolateral nuclei, and adds another distinction: olfactory connectivity. The MeA, ACo, and PAC exhibit preferred connectivity with the OB in humans, and therefore likely have unique functions in odor processing in humans. Detailed future studies of each amygdala subregion are needed in order to fully understand the function of the human olfactory amygdala.

## 5 Limitations

Our study has several limitations, including a relatively small sample size and the fact that we used the olfactory tract as the seeding point to work around the technical difficulty of direct streamline tracing from the olfactory bulb. Our use of hand-drawn ROIs may hamper reproducibility of our results, and to address this limitation, we have provided detailed ROI drawing instructions inSection 2, and have made our hand-drawn ROIs publicly available. We also note that dMRI methods carry inherent limitations, including the inability to determine directionality of anatomical connections, or to determine the number of synapses between target regions. Therefore, our findings cannot be interpreted as conclusive evidence for direct, monosynaptic connections between the olfactory bulb and the amygdala in humans. We also note that evidence from diffusion tractography alone is not enough to discover new facts about brain connectivity, but only to confirm with non-invasive imaging facts that have been previously shown with invasive anatomical studies. Our work here is not identifying new anatomical projections, but rather provides in-vivo verification of post-mortem silver-staining work fromAllison (1954), which identified projections from the human olfactory bulb to amygdala subregions.

## Supplementary Material

Supplementary Material

## Data Availability

Data will be shared for specific projects under a formal data-sharing agreement.
