# Supplementary material for: The human olfactory amygdala: Anatomical connections between the olfactory bulb and amygdala subregions
Source: Imaging Neurosci (Camb). 2025 May 9;3:imag_a_00571. doi: 10.1162/imag_a_00571 (PMC12319816; doi:10.1162/imag_a_00571)

Supplementary data:

Supp. Fig. 1: Cortical structural connectivity with CeA included in olfactory subregions

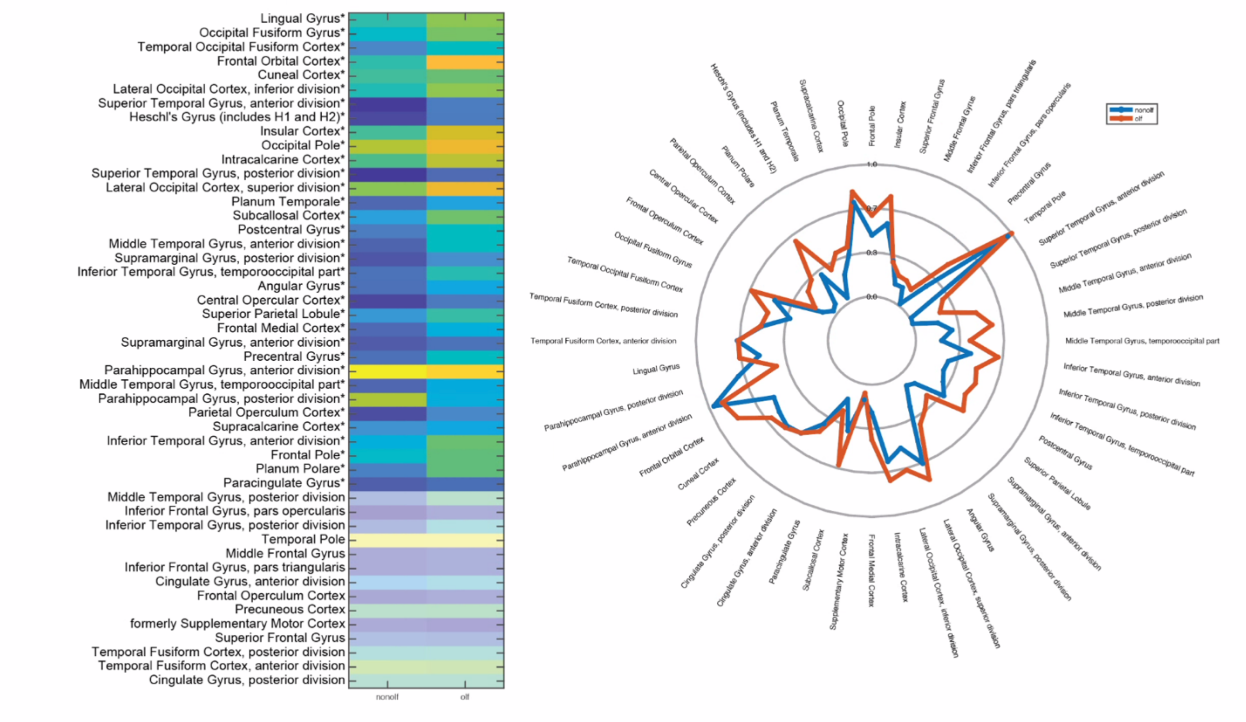

Supp. Fig. 2: Subcortical structural connectivity with CeA included in olfactory subregions

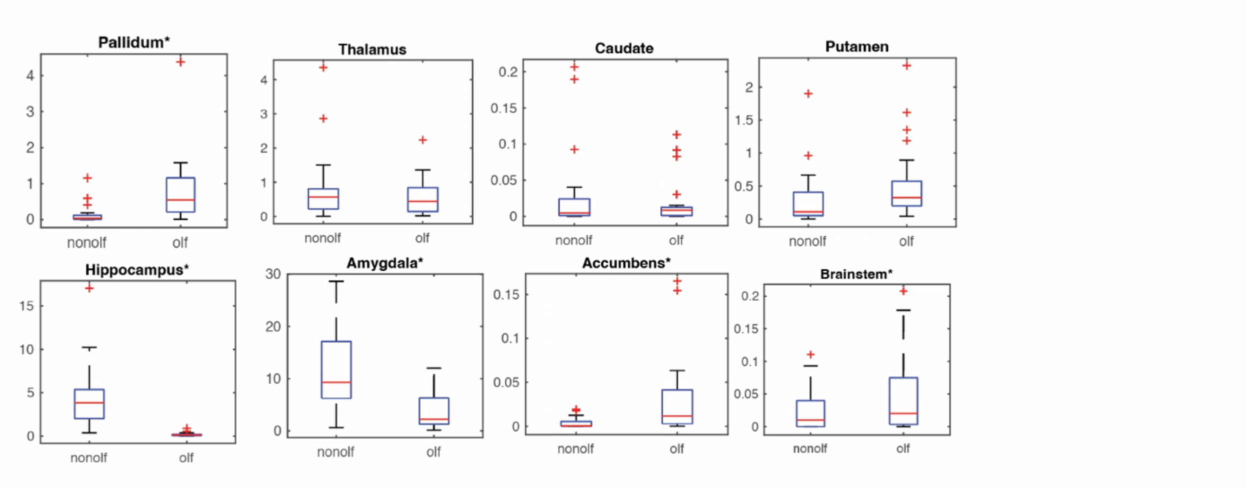

Supp. Fig. 3: Using distance as the measurement for k-means clustering

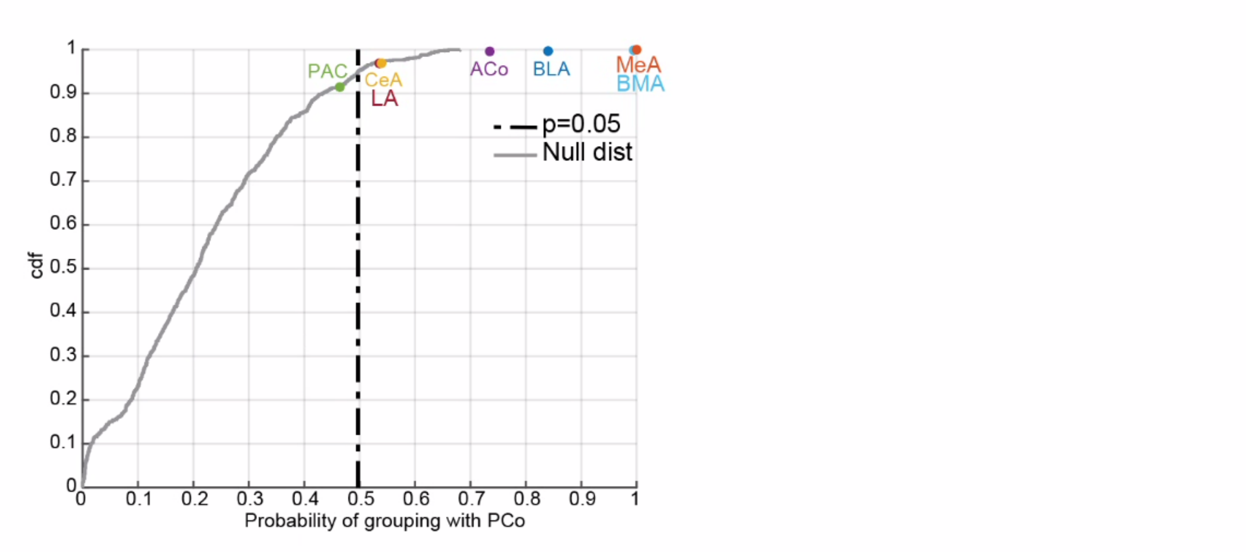

Supp. Fig. 4: Correlation analysis between structural features and olfactory perception

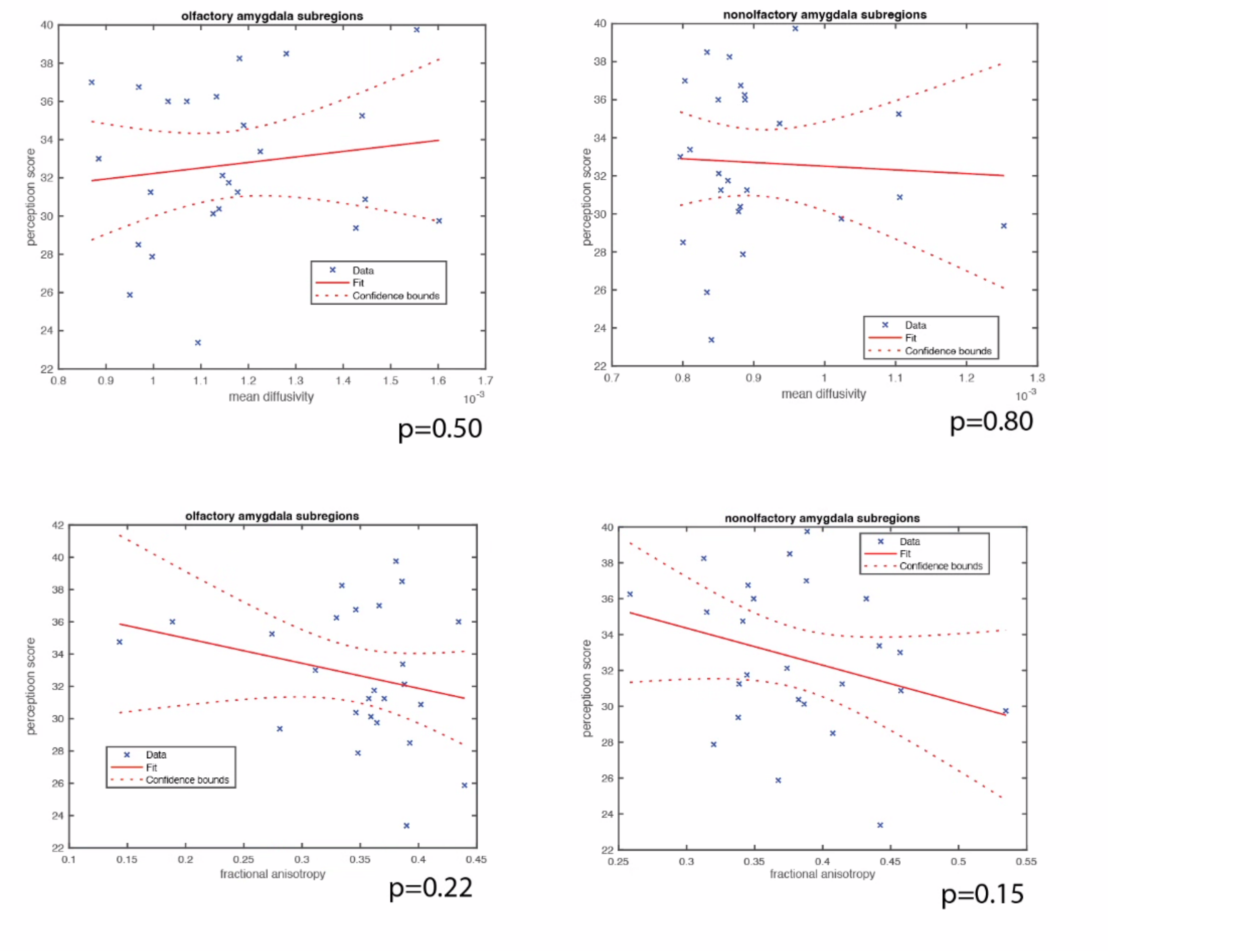

Supplement: Supplementary Material [file imag_a_00571-supp.pdf]
